# Protecting the Bunce Legacy: Lessons Learned From Safeguarding Long-term Ecological Survey Datasets in Great Britain

**DOI:** 10.1007/s00267-025-02175-5

**Published:** 2025-04-25

**Authors:** Claire M. Wood, Marc J. Metzger, Robert G. H. Bunce

**Affiliations:** 1 UK Centre for Ecology & Hydrology, Library Avenue, Lancaster, UK; 2 School of GeoSciences, University of Edinburgh, Edinburgh, UK; 3 Estonian University of Life Sciences, Kreuzwaldi 5, Tartu, Estonia

**Keywords:** Long-term ecological monitoring, data rescue, legacy data, data curation, data accessibility

## Abstract

Rescued data helps to strengthen ecological understanding of biodiversity change. This paper presents experience from safeguarding long-term strategic ecological surveys established by the late Professor Robert Bunce and colleagues in the 1970s: the Great Britain Countryside Surveys, and various related and complementary surveys in the period 1969 to the mid-1990s, including woodland surveys, and regional surveys for Cumbria and Shetland. These surveys are valuable data sources - especially considering national and global ecological restoration targets to address the biodiversity crisis - providing evidence to explore and understand ecological changes in the British countryside over time. For these kinds of data to be useful, usable and used, it is essential they are accessible and well managed, but many important ecological data sets are at risk of loss. A decade of work to protect the Bunce surveys has resulted in a structured five-step approach that can benefit other data rescue and safeguarding initiatives as well as scientists planning new ecological monitoring projects. The steps involve identifying available resources, processing datasets, assembling metadata, producing outputs and publishing. Valuable lessons learnt in the process include: (1) the growing appreciation and relevance of historic ecological data; (2) the importance of adequate resourcing and recognition of data rescue activity; (3) the value of engaging with the originators; (4) the need to identify and understand potential users and uses of the data. The Bunce legacy of strategic ecological surveys in the UK is now protected and the data available for repeat survey and further analysis.

## Introduction

Long-term ecological monitoring provides crucial data to understand the current global biodiversity crisis (IPBES 2019a; Scholes et al. 2012) and confidently review progress towards the conservation and restoration targets set out in the Kunming-Montreal Global Biodiversity Framework (CBD 2022; GEO BON 2022; Gonzalez et al. 2023; IPBES 2019b). A useful definition of ecological long-term monitoring is provided by Lindenmayer et al. (2012) as ‘the systematic and regular collection of field data from a particular site or set of sites for more than 10 years’. High-quality ecological information collected over long periods yields valuable insights into changes in ecosystem structure, key ecological processes and the services provided by ecosystems (IPBES 2019a), and inform actions to restore nature (Paolinelli Reis et al. 2024). Frustratingly, while the value of long-term data collected historically is increasingly appreciated, data are being lost at a rapid rate, for example, due to a lack of management or mistaken discard. When they do still exist, they are often impossible to access, either due to being stored on old formats or because few people know they exist (Griffin, 2017).

In 2013, it was estimated that 80% of all ecological data used in papers dating back to as recently as the 1990s has been lost (Gibney and Van Noorden 2013). We follow Wilkinson et al. (2016) by considering data ‘lost’ when they are not Findable, Accessible, Interoperable and Reusable (FAIR) because they are not in a usable state, and therefore are liable to being forgotten about and possibly then discarded. A prime example of data from a large programme being ‘lost’ is the International Biological Programme (IBP) (Worthington 1965), which has failed to have a lasting legacy because the data collected were not made available for re-use (Hampton et al. 2013; Michener et al. 1997). As Griffin (2017) expressed: ‘Treasure troves of data, and the knowledge they could offer, are left mouldering on shelves’. Such datasets failing to meet multiple FAIR criteria can be considered ‘at risk’. Safe-guarding datasets requires an ongoing process of preserving data at risk of being lost. This process might involve digitising current and past data into computer-compatible form for easy access (Diwakar et al. 2008). If this process is not maintained, ‘data rescue’ becomes necessary to prevent the data (and accompanying metadata) being lost forever. Data rescue comprises the concerted and resource intensive effort to ensure legacy data meets the FAIR criteria (Wilkinson et al., 2016). As old storage media become unusable, accidents such as fire, flood and mistaken discard occur, and the original data collectors are no longer available to question, the opportunities for rescuing many datasets are fading (Downs and Chen 2017; Griffin 2017; Whitlock 2011).

**Fig. 1:**
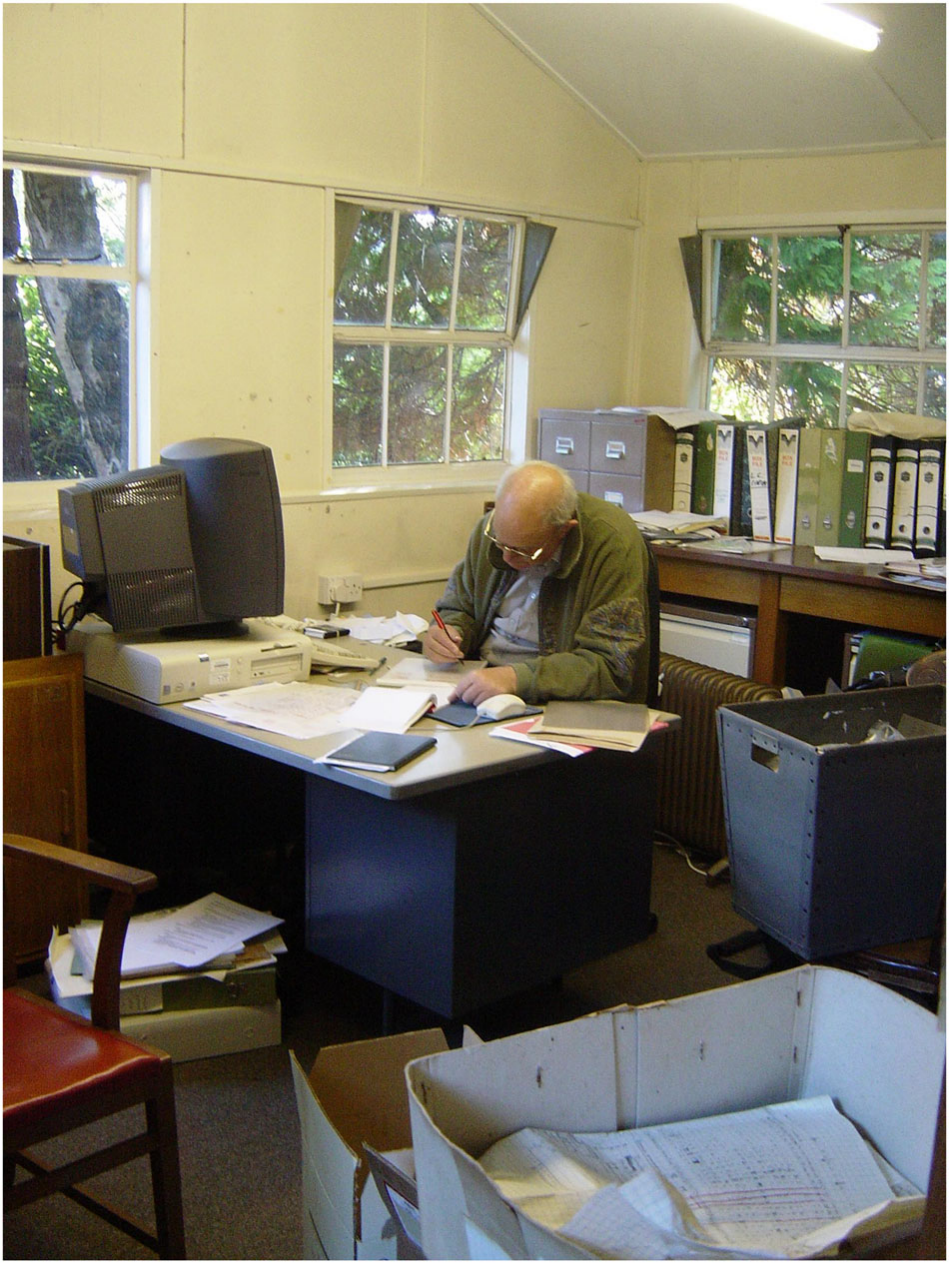
Professor Bunce at work at Merlewood Research Station, circa 2000

This paper focuses on the safeguarding of the Bunce datasets (see Online Resource material), which in most cases included data rescue. In the late 1960s, driven by a policy interest in objective measures to understand the decline of vegetation and habitats in Great Britain, Robert (Bob) G.H. Bunce (Fig. 1) and colleagues at the Nature Conservancy (now part of the UK Centre for Ecology & Hydrology (UKCEH)) laid the basis for a series of strategic ecological surveys (Sheail & Bunce, 2003). These surveys, described in section 2, provide statistically robust, repeatable surveys of vascular plants, trees, soils and landscape features and include the Countryside Survey of Great Britain (GB), running since 1978 (Bunce 1979; Norton et al. 2012a) and the Bunce Woodland Surveys (Bunce and Shaw 1980; Kirby et al. 2005; Smart et al. 2024), running since 1971 (Sheail and Bunce 2003). The repeatable methods and ethos of all the Bunce surveys have inspired other monitoring programmes, some international, for example in Northern Ireland (Cooper et al. 2009), Wales (Wood et al. 2021), Sweden (Ståhl et al. 2011) and Norway (Dramstad et al. 2002) and a pan-European habitat monitoring design (Metzger et al. 2013). Potential users of the Bunce data are wide-ranging, from policy-makers to researchers and students. The data are useful for anyone who needs to understand environmental change, and to answer questions about why those changes are occurring, and how best to manage those changes.

Whereas the more recent GB Countryside Survey datasets have been continuously maintained, nine historic Bunce datasets were, until recently, in danger of being lost forever due to non-accessible data formats and structures, and missing metadata. Michener et al. (1997) proposed the idea of ‘information entropy’ in relation to ecological sciences, whereby metadata - the “who, what, when, where, and how” about every aspect of data - degrades over the lifespan of the originator. With Professor Bunce’s death in April 2022 (Howard 2022), we have now sadly reached the endpoint of the information entropy of the Bunce datasets. However, thanks to a major data rescue and safeguarding operation begun in the 2010s, the Bunce legacy is now secure for the long term. Here, we describe the five-step data rescue approach developed to rescue the Bunce ecological surveys and reflect on wider lessons learned for conserving ecological survey data.

## The Bunce Strategic Ecological Surveys and Their Data

### The Bunce Surveys

In Britain, early ecological survey approaches focused on recording only the dominant species and the general state and condition of plant communities (Moss 1910; Tansley 1939) or classified vegetation using characteristic species (Braun-Blanquet 1932; Poore 1955). These descriptive, qualitative approaches relied upon the surveyors’ variable judgements and were criticised by ecologists in the 1950s for lacking an exact basis (Webb 1954). Emerging computational and statistical techniques opened possibilities to moving towards standardised sampling designs and field protocols that would enable changes in species composition over time to be measured and quantified objectively. In collaboration with leading quantitative ecologists and statisticians, Bunce developed statistically robust, standardised, repeatable sampling methods for producing estimates of ecological metrics at landscape scale for large areas using stratified random samples (Sheail and Bunce, 2003).

Early surveys in British woodlands, followed by regional surveys in Shetland and Cumbria, led to the initiation of the first national ecological survey of Great Britain (GB) (an area of over 209,000 km^2^) in 1978, the GB Countryside Survey (Bunce 1979); Table 1). The GB Countryside Survey has been repeated in 1984, 1990, 1998 and 2007 (Barr et al. 1986; Barr et al. 1993; Firbank et al. 2003; Norton et al. 2012a) and in 2019, became a ‘rolling’ survey whereby a subset of the total survey sites are visited each year over a five-year period, currently focusing on soil sampling (analysis of physicochemical properties) and vegetation (plant species recording) only. Now Britain’s longest running large-scale ecological monitoring programme, a unique strength of the GB Countryside Survey has been that, over time, data have been simultaneously collected from the same 1 × 1-km sample square from permanently sited vegetation plots with integrated landscape scale habitat mapping, soil samples (physicochemical properties, microbiology, invertebrates), and freshwater sampling (plant and macroinvertebrates, pond inventories, water chemistry) (UKCEH 2024). This landscape scale approach allows linkages to be made between the causes of change over a 35+ year period in land-use, vegetation, soil and freshwater (NEA UK 2011; Norton et al. 2012a; Seaton et al. 2020; Spake et al. 2019). The 1971 Woodland Survey has also been repeated in 2002 and 2022 (Kirby et al. 2005; Smart et al. 2024; Smart and Wood 2021). The development and scientific principles of the Bunce surveys for strategical ecological surveys are described in detail by Sheail and Bunce (2003). Table 1 provides an overview of the most important Bunce surveys, with additional details and surveys included in the Online Resource material.

**Table 1:** Overview and chronology of the key Bunce surveys, building on the overview provided by Sheail & Bunce (2003)

*Date and project*	*Characteristics*
**1971–2022 Woodland Survey (**Wood, Smart and Bunce 2015**)**	• 2453 woodlands in UK defined for survey cartographically • Limited species lists classified statistically into 103 groups of woodlands • Analysis of environmental variables from the woodlands • 103 sites selected at random from the environmental analysis • 16 random dispersed 200 m square vegetation plots per wood • Classification using ISA (TWINSPAN) into 13 site classes and 32 plot classes • Interpretation of the site and plot vegetation classifications
**1971–2022 Scottish Pinewoods Survey (**Wood and Bunce 2016b**)**	• 27 pinewoods in Scotland • 16 random dispersed 200 m square vegetation plots per wood • Interpretation of the plot and site classifications
**1974 Shetland Ecological Survey (**Wood and Bunce 2016a**)**	• Environmental data from 2046 1-km squares on a 1-km grid in Shetland • Classification by ISA (TWINSPAN) into 16 land classes • Five random 1-km squares drawn from each class • Up to 16 dispersed random 200 m vegetation plots classified by ISA (TWINSPAN) into 16 vegetation classes • Interpretation of the characteristics of the land classes and vegetation classes
**1975 Cumbria Ecological Survey**	• Environmental data from 850 1-km Ecological Survey squares on a 3 × 3 km grid in Cumbria • Classification by ISA (TWINSPAN) into 16 land classes • Remaining 6650 1 km squares in Cumbria classified by key attributes • Three random 1-km squares drawn from each class • Eight or 16 dispersed random 200 m square vegetation plots classified by ISA (TWINSPAN) into 16 vegetation classes • Interpretation of the characteristics of the land classes and vegetation classes
**1978-present** **Great Britain Countryside Survey (**Wood et al. 2017, 2018a**)**	• Environmental data from 1228 1-km Great Britain squares on a 15 km grid in UK Countryside • Classification by TWINSPAN into 45 Survey land classes • Allocation by a statistical routine of the remaining c. 233,000 1-km squares • In 1978, 256 1-km squares drawn at random, increasing progressively to 591 1 km squares in 2007 • Up to 42 vegetation plots per 1-km square, mapping of broad habitats and records of other ecological data • Classification by TWINSPAN into 100 vegetation classes • Integrated interpretation of resources and change of broad habitats, soils, vegetation classes and vascular plants
**1992–93 Survey of ‘Key Habitats’ in England (**Wood et al. 2018b**)**	• Environmental data from 4 spatial masks in England (Calcareous, Coastal, Upland & Lowland Heath) • 213 1-km squares drawn at random • Up to 25 vegetation plots per 1-km square • Integrated interpretation of resources and change of habitats, vegetation classes and vascular plants

Additional detail, including for smaller surveys, can be found in the Online Resource material

Since the early Bunce surveys, ecological status and trends have been assessed by other field-based monitoring programmes (e.g., the National Plant Monitoring Scheme (NPMS, 2024) and the UK Environmental Change Network (UKECN 2024) and through earth observation (UKCEH Land Cover Maps 2024, Broughton et al. 2024). While each approach has its specific merits and objectives, Table 2 illustrates how the Bunce surveys pre-date other studies by more than a decade providing a substantially deeper time window for understanding change. Furthermore, Table 2 demonstrates that the Bunce surveys are unique in their ability to simultaneously address a suite of policy-relevant environmental quality indicators, identified by Carey et al. (2008). For example, the GB Countryside Survey provides measures of Broad Habitat areas at a national level dating back to 1978, including change in those areas, measures of condition within those habitats, and the condition of vegetation (such as species richness, and the number of butterfly food plants). It also provides an estimate of linear landscape features (perhaps most importantly, hedges) and data regarding pond quality and quantity, and soil properties such as pH and carbon measures.

**Table 2:**
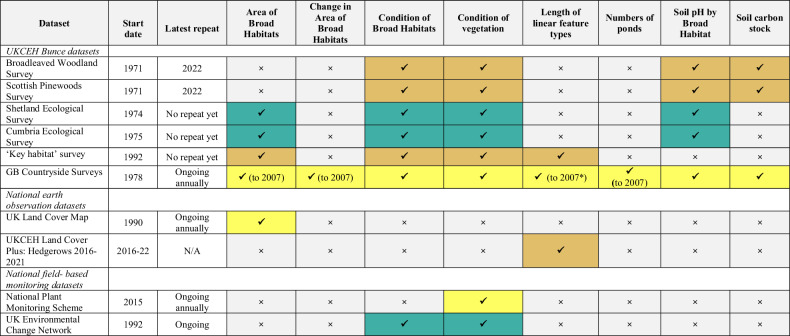
Comparison of the Bunce surveys with other national programmes assessing ecological status and trends in the UK for a range of policy-relevant indicators of environmental quality proposed by Carey et al. (2008)

^*^Hedgerows were surveyed in England in 2022/23

### Survey Data

**Fig. 2:**
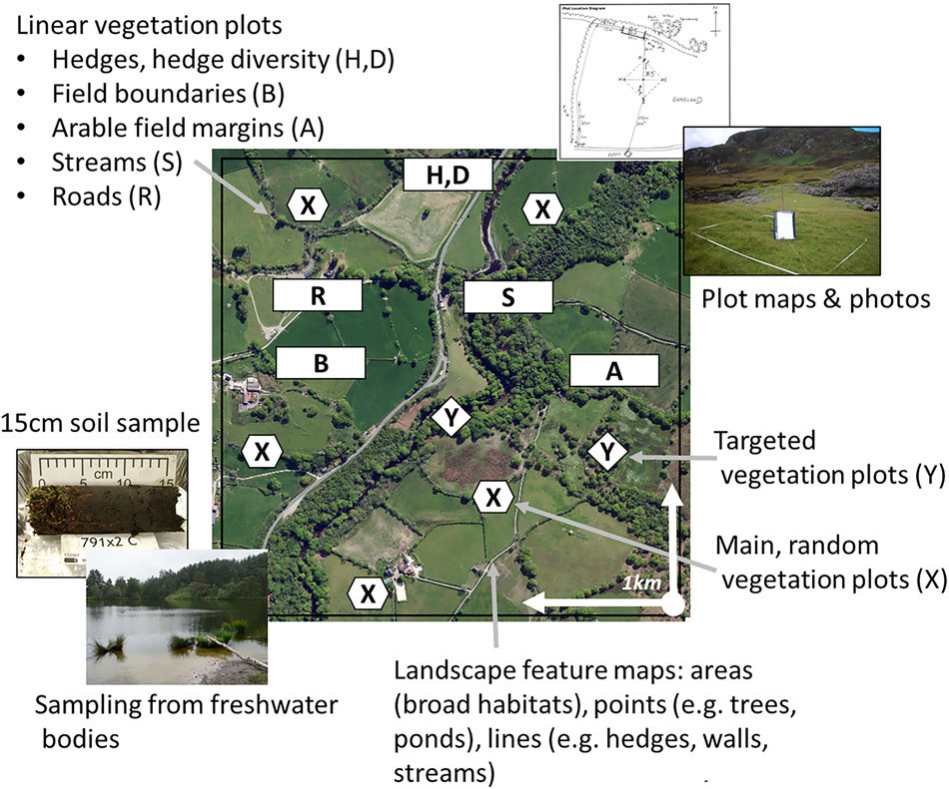
Example of a 1-km survey square and the types of data collected during surveys

The Bunce surveys all follow a similar methodological approach and design, as introduced in the 1970s: (1) a land classification or stratification of the survey area in question (based on geology, climate and geographical features), be it Shetland, Cumbria, GB, or woodlands to ensure the survey sample was representative of the range of different environments; (2) selecting random sample sites for survey, usually sized 1 × 1-km, referred to as ‘1-km squares’, within each land class; and (3) strict protocols to record a variety of ecological parameters according to the landscape configuration at the site (e.g. linear features, vegetation, species, soil and water properties) within each site or 1-km square. This sample design allows estimates, and their uncertainties, to be calculated for each stratum and the entire geographic region (e.g., all of GB) using standard statistical inference (Metzger et al. 2013). Each survey results in a collection of associated datasets relating to the sample design (the stratification and sample locations) and each 1-km square or site; records of field recordings (vegetation, species, soil and water properties), maps, locations of sample plots, and photographs and field notes. An example of a 1-km survey square is shown in Fig. 2. This example is most similar to a GB Countryside Survey 1-km square but illustrates the diverse types of data that may have been collected during different Bunce surveys.

### Data Rescue Challenges

All of the UKCEH Bunce surveys were designed with the possibility of repeats taking place. However, due to funding and resource issues, repeat surveys were never guaranteed. With hindsight, it is notable that the survey on the scale of the GB Countryside Survey has now been repeated five times since 1978 and is still ongoing. It is easy to look back and take the continuity and repeatability of the GB Countryside Survey for granted. However, several of the historic Bunce surveys have still not been repeated (Shetland, Cumbria, and a survey of ‘Key Habitats’ - a term included in the UK biodiversity action plans (UK Biodiversity Steering Group, 1995), which were later to evolve into the Broad and Priority Habitat framework). Others took many years to accomplish: the first re-survey of the Woodland Survey took 30 years; and the first re-survey of the Scottish Pinewoods was not fully repeated until 50 years after the original survey). Aside from resourcing issues, one reason for this has been the lack of availability and usability of the original raw data and documentation, and the challenges associated with making them useful for the purposes of repeating a survey. In short, they were at risk of being lost by failing multiple FAIR criteria. There are many threats to ‘at-risk’ data (Patterton et al. 2024). Below, we summarise the main challenges encountered during the safeguarding of the historic Bunce surveys, carried out between 2010 and 2020.

#### Skills and Resource Needs

The immediate priority for the Bunce surveys has always been an emphasis on collecting new data and publishing new results to disseminate the latest key messages regarding environmental state or change. Until recently, the importance of curating historic data was not recognised and resources were not made available to undertake such a large task, nor were the tools available to do so. Fortunately, the paper records have been kept safely in secure storage, with associated survey documents. Furthermore, the scientific originator, Bob Bunce, was available to pass on information, clarify and explain the documents and any anomalies.

A lack of proper data management, including time for database development, data entry, data validation, analysis, interpretation and reporting of data can be one of the reasons why long-term monitoring programs fail (Caughlan and Oakley 2001). The nature of the surveys in question means that the data collected is wide ranging (cf. Fig. 2) and complex in terms of data management. Overall, each survey follows similar and standardised protocols, which makes the task of understanding the data structures slightly easier across the surveys. However, a specific challenge was finding the correct descriptions for recorded codes (e.g. plant species) and applying those consistently, understanding relationships between different groupings of data (for example matching plot and site codes from different tables), and being able to organise the data sensibly within a relational database to optimise re-usability of the datasets.

#### Technological Change

**Fig. 3:**
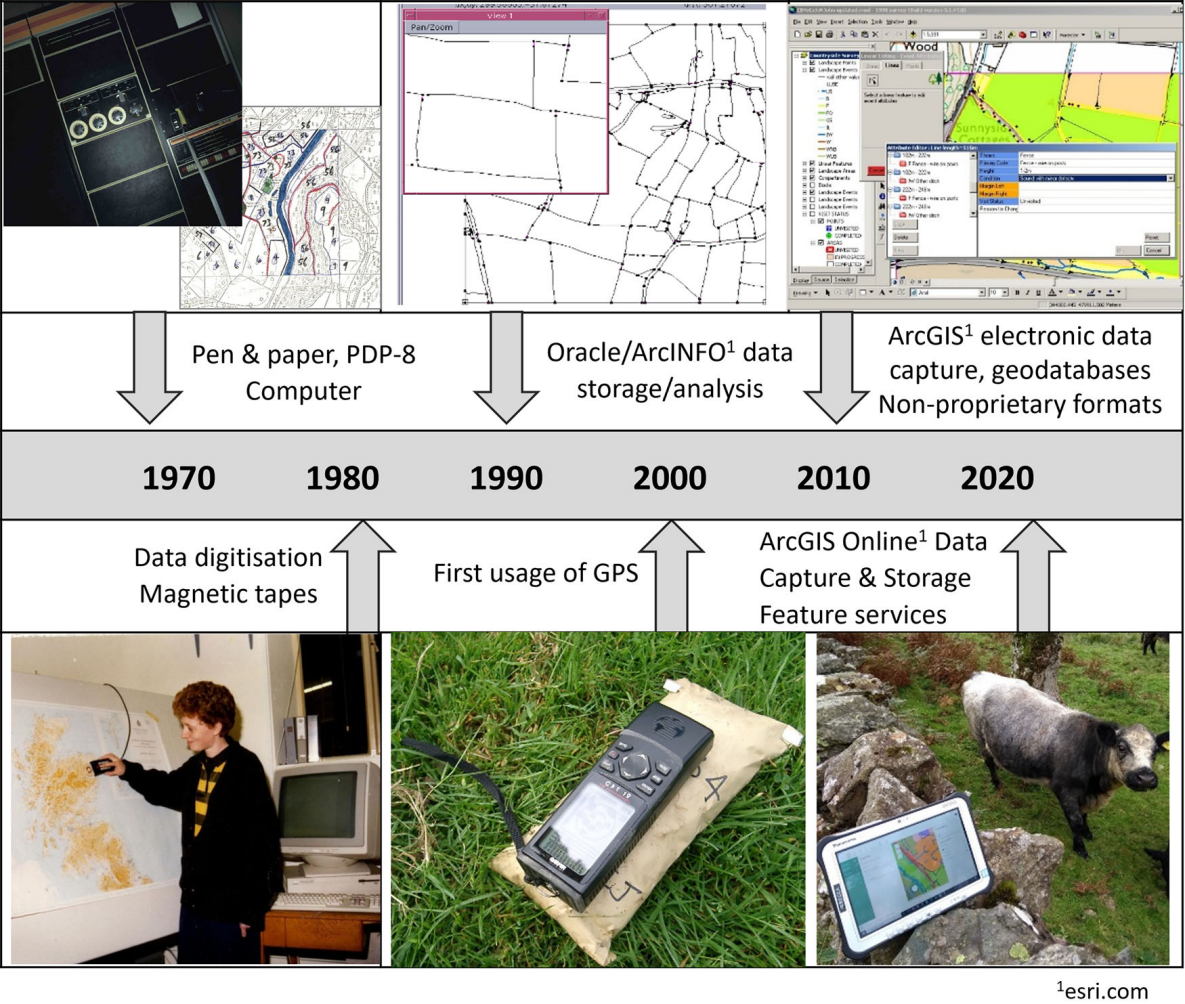
Technological developments during the Bunce surveys

Data storage technology has advanced rapidly over the last decades, which is reflected in the storage media used for the Bunce surveys (Fig. 3). Much of the earlier survey data were stored on tapes used by a PDP-8 computer, which did not stand the test of time as they can no longer be read by readily available equipment. Luckily, the early Bunce surveys were carried out using paper recording sheets, which had been archived and could be digitised once more, despite the digitisation process being challenging. Re-digitising takes time, and involves deciphering handwriting, old codes and protocols and with no access to the original surveyors. Re-digitising does mean that the data can be structured in an optimal way to encourage reusability, and to be stored in such a format as to be acceptable to a long-term data repository, and fortunately, Bob Bunce was on hand to assist with clarifying procedures and methods, and with deciphering his notorious handwriting.

Later surveys (for example, the ‘Key Habitats’ survey; Table 1) made use of emerging technologies, including the use of Geographical Information Systems (GIS) and Oracle (www.oracle.com) relational databases. Software and infrastructures for managing spatial data have constantly evolved in terms of functionality and ease of use, and knowledge regarding the most durable formats for storing data for the long-term have advanced considerably. Despite this, over time it is still possible for data to become obsolete, if not continuously maintained in the latest or non-proprietary versions. These insights have grown over time, and guidance for long-term electronic storage is available, for example from the Digital Data Curation Centre (https://www.dcc.ac.uk/).

#### Data Storage Infrastructure

In practical terms, the idea of public data archives and repositories to deposit (ecological) data and metadata is relatively new. Before these repositories were in place, there was nowhere to store (legacy) data long-term (Michener 2006). The historic Bunce surveys have relied on paper storage for their longevity. A major driver for the establishment of the UKs National Environment Research Council (NERC) Environmental Information Data Centre (EIDC) (https://eidc.ac.uk/), which hosts publicly funded data relating to terrestrial and freshwater sciences, was an increasing need for the dissemination, long-term storage and documentation of long-term monitoring datasets arising from UKCEH work, and particularly the GB Countryside Survey.

Thanks to technological advances, practical solutions for long-term data storage and security are now available. There is also a wider appreciation of the importance of a need for careful data management planning when undertaking publicly funded scientific work, which helps scientists consider the type and amount of data to be collected, long-term data storage plans, metadata and documentation, licensing and copyright during the project design.

#### Privacy and Data Protection

Site locations of the Bunce surveys have always been held confidentially, most strictly in the case of the GB Countryside Surveys. Because surveys are largely carried out on private land, they rely on access permission and it is therefore important that landowners relations are managed carefully, as ‘survey fatigue’ could compromise future surveys. Landowners may not want to publicise the presence of, for example, certain plant species on their land or welcome an increase in visits from interested parties. Additionally, due to the statistical design of the surveys, it is important to avoid the introduction of bias into the sample, which could happen if a landowner could identify the poor ecological status of their land and decide to address this based on the survey information (e.g., by liming acidic soils). Therefore, preserving the representativeness of sampling sites and the goodwill of landowners are both essential elements to the future of surveys to ensure the scientific integrity of the sampling strategy, the protection of the environment, and help to ensure future permission from landowners to survey their land. While restricting access to the specific site locations raises a barrier to truly ‘open data’, the restrictions do not preclude the re-use of the data, as data are available at greater spatial resolutions, excluding any personal information. Due to the long-term nature of the work, the risk of compromising future surveys must be minimised.

### A Five-Step Approach to Safeguarding and Rescuing Ecological Survey Data

**Fig. 4:**
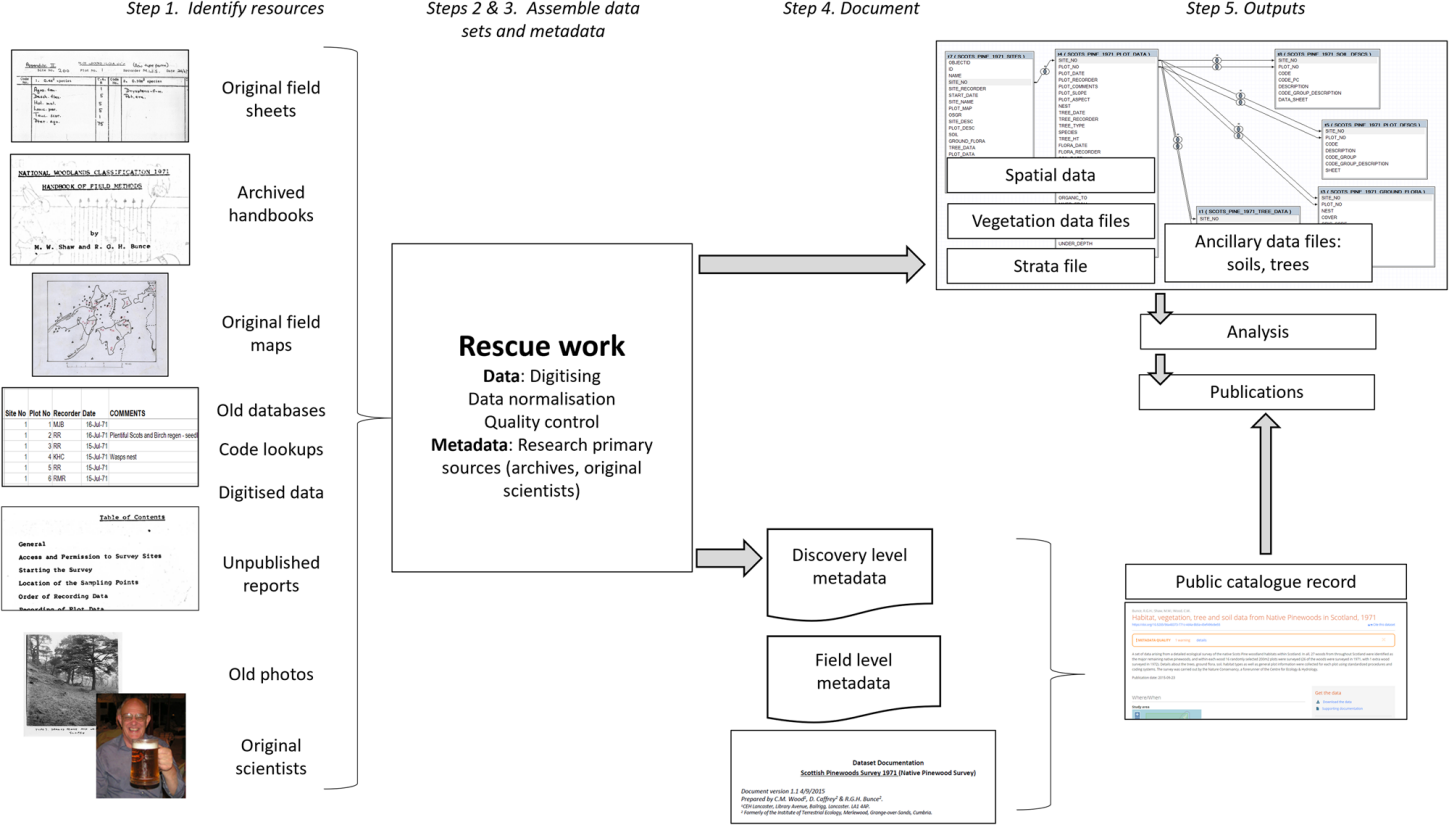
Data rescue flow chart, with examples from the Bunce surveys

Experience gained during approximately 10 years of safeguarding the Bunce survey data, resulted in a general five-step approach to safeguarding and rescuing ecological survey data that are considered to have long-term value (Table 3; Fig. 4). Depending on the status of the dataset in question, it may be possible to bypass certain early steps. We describe generic steps, illustrated with experience and examples from our work, as appropriate to the rescue of the Bunce legacy datasets.

**Table 3:** Overview of the five steps to rescuing ecological survey data, with a summary of the activities involved to safeguard the Bunce datasets

Step	Activities undertaken to safeguard the Bunce datasets
1. Identify available resources	**• Search** for existence of: archived field handbooks; paper recording sheets; code lookups; paper field maps; old photos; previously digitised data in spreadsheets; data in old databases; published and unpublished reports **• Discuss** surveys and data collection with original scientists
2. Process datasets	**• Plan** a data schema to store data **• Digitise** analogue data and reformat digital data to open data formats **• Structure** data to for optimal retrieval and re-use **• Quality Assurance and Control** checks for any missing data and inconsistencies
3. Assemble metadata	**• Who** were the surveyors? **• What** do recorded codes mean? **• Where** are the sample locations? **• When** were the data collected? **• How** were the data collected?
4. Produce outputs	**• Datasets** comprising: a strata file; vegetation records; soil records; tree records; sampling locations; spatial landscape data and other plot or site-based descriptions. **• Metadata documents** describing the information uncovered in steps 2 and 3
5. Publication	**• Citable datasets** with Digital Object Identifiers (DOIs) **• Data publication** in an academic journal (optional) **• Data analysis** revisiting the historic data (optional)

### Step 1: Identify Available Resources

Perhaps self-evident, but identifying and locating the available resources relating to the dataset in question is an important first step. This includes paper documents such as field handbooks, field recording sheets, photographs, maps and code descriptions. In some cases, there may also be extant digitally recorded resources. For the Bunce surveys, resources were found in the UKCEH archives, staff offices, a fire safe, network drives, and in the case of one set of maps, a garden shed.

### Steps 2: Process Datasets

Data may have to be digitised or converted to an appropriate data format. At this stage, for optimal storage efficiency of complex data, a sensible data schema should be designed, providing a plan how datasets and data elements will be named, structured and related within a relational database (for example, stored in MS Access or a corporate system such as Oracle). The data must then be structured, or possibly converted, to fit into the planned schema. For the Bunce surveys, the final archived datasets consisted of some or all of the following data elements: a strata file, vegetation records, soil records, tree records, sampling locations, spatial landscape data, and other plot or site-based descriptions including photographs. Each dataset is linked by either the plot or site codes to sampling locations. Whilst a relational database is helpful for efficient internal storage and retrieval of data, data may be made available in a more user-friendly, non-proprietary format (for example, comma-separated value files) as described in Step 4.

### Step 3: Assemble Metadata

Steps 2 and 3 may be iterative, as organising the data relies on some knowledge of the metadata, but also constructing metadata also relies on knowledge of the data. Whilst working with the data, questions are likely to arise concerning the nature and units of recorded values, field methods and experimental design. Answering these questions with the assistance of available documents and the personal knowledge of the original scientist is essential to construct adequate metadata to accompany the dataset. At this stage, the data may be stored in an internal file storage system or database, potentially in a proprietary format. For the Bunce surveys, useful documents in helping develop supporting documentation included field handbooks (e.g., Barr 1992), photographs and site maps, and personal insight from the initiator of the projects.

### Step 4: Produce Outputs

This step concerns the creation of re-usable and publishable data products and includes both the dataset itself, presented in a robust, non-proprietary format, and the explanatory metadata in a comprehensive, user-friendly document. An example of these can be downloaded from the landing page for the Shetland Survey at 10.5285/06fc0b8c-cc4a-4ea8-b4be-f8bd7ee25342.

### Step 5: Publication

The final stage concerns the publication of the data products in a data repository and obtaining a Digital Object Identifier (DOI) and citation for the dataset. There are various free and chargeable data repositories, including Dryad (https://datadryad.org/), figshare (https://figshare.com/), Zenodo (https://zenodo.org/), including many thematic repositories (e.g., the Marine Geoscience Data System, https://www.marine-geo.org/index.php). The publication stage may also reference, analyse or describe the published dataset in a peer-reviewed scientific or data journal. The Bunce surveys were deposited in the NERC EIDC (https://eidc.ac.uk/*)* as the UK’s national data centre for terrestrial and freshwater sciences. A full list of the Bunce related datasets and papers is included in the Online Resource material.

## Discussion

### Opportunities and Challenges

#### Legacy Data Can Advance Understanding of Ecological Change

Recognition of the economic and societal consequences of the biodiversity crisis (de Oliveira Caetano et al., 2023; IPBES 2019b), corporate biodiversity risk exposure (Carvalho et al. 2023), and legally binding nature of halting biodiversity loss (Ekardt et al. 2023) have resulted in – and focused attention on – the ambitious targets of the Kunming-Montreal Global Biodiversity Framework (CBD 2022). A significant development is the focus on reversing loss and achieving biodiversity gain, placing an even greater emphasis on understanding and quantifying ecological change, including interest from emerging biodiversity markets (Lindenmayer et al. 2023). Rescued data helps to strengthen ecological understanding of biodiversity change by providing ‘big data’ for meta-studies (Lindenmayer et al. 2012; Pilotto et al. 2020; Thackeray et al. 2010) and ecological models (Henrys et al. 2015a; Henrys et al. 2015b; Pollock et al. 2020), increasingly so considering the ability of Artificial Intelligence to extract learning from large and diverse data collections (Christin et al. 2019; Ryo 2024). And in the case of ecological survey data, rescued data can form the basis for repeat surveys, establishing true measurements of ecological change over time (GLORIA 2024; Lodetti et al. 2024; Rich et al. 2015; Riedel et al. 2023; Wood et al. 2015). But, despite huge improvements in the ability to manage and store data from contemporary research, there has been little concerted effort to progress ecological data rescue since Hampton et al. (2013) reflected that there remain many old boxes of nationally important information sitting on shelves, or on obsolete storage media. Considering the urgency and importance of the biodiversity crisis, the potential of these legacy sources to advance ecological understanding deserves greater consideration.

#### Data Rescue Requires Adequate Resourcing and Appreciation

Data rescue and safeguarding tasks are rarely as simple as just digitising analogue data. It takes time, dedication, and a specific skillset that straddles ecological, data, and archival sciences to uncover these data. Griffin (2017) noted that few want to ‘poke around musty archives for heritage data captured using yesterday’s technology’. Uncovering past achievements is less prestigious than leading new academic discovery, which is reflected in limited institutional and research funding to support data rescue, storage and archiving and little credit and esteem is given to those involved in the process. Despite these challenges, the drive for ‘open data’ (Open Data Handbook 2024) has provided the infrastructure to share legacy data through repositories and data journals, increased the general appreciation of data sharing, and provided opportunities to credit the work through citations. Open data can be defined as ‘data that anyone can access, use and share’ (Open Data Institute 2017). To be strictly ‘open’, data should be completely free, with no restrictions (Attard et al. 2015). Advantages of open data include the ability to find and re-use data, enhancing opportunities for collaboration and meta-analysis, and the prospect to answer questions not previously posed by the original data collectors. In our case, data rescue was not considered a priority in terms of funding and resources, and required many hours of the personal investment of time over a decade. Increasing data rescue effort will require research funders and employers to more explicitly appreciate and value these efforts.

#### Data Rescue Requires Structured Approach

Data archiving infrastructure is now commonplace, along with associated standards for data archiving and metadata reporting (International Organization for Standardization 2015; UK GEMINI 2015) but good practice guidelines how to approach data rescue have not been readily available until recently (Bledsoe et al. 2022; Patterton et al. 2024). Our five-step approach (Fig. 4 and Table 3) was developed in tandem with the inauguration of the NERC EIDC in 2012. The EIDC and associated guidance for storage and publication, were themselves informed by lessons learned from the ongoing data management procedures involved with the GB Countryside Survey datasets, and an awareness that the historic datasets were ‘at risk’ and action needed to be taken. While our approach was developed and tailored for the Bunce datasets, it is adaptable and can form the basis for a structured approach for other data rescue and archiving initiatives. Important lessons from our experience, reflected in the five-step approach, include the importance to take time to engage with the originators at an early stage to understand the background and value of the data, and to take time to identify and understand potential users and uses of the data.

### The Bunce Legacy

For over 40 years, the strategic ecological survey methods developed by Bob Bunce and colleagues have provided robust information about the state of Britain’s countryside, providing statistical estimates of the decline in vegetation and habitats (Sheail and Bunce, 2003). Metzger et al. (2013) identified the following reasons behind the enduring success of the approach: (1) the use of environmental stratification to ensure representativeness; (2) using probability sampling to provide robust statistical estimates of change; (3) adopting a flexible sampling design that allowed for adding new samples or losing sample units that could no longer be reported; (4) collecting disaggregated data to allow construction of aggregated indicators tailored to the most recent policy questions; and (5) collecting species level data to understand change in habitat quality.

In the foreword of the 2000 GB Countryside Survey report, the UK Minster for the Environment, Michael Meacher, wrote ‘good management requires reliable information to measure progress, stimulate debate and inform decisions’ (Haines-Young et al. 2000). By that time, measurements by the GB Countryside Survey had quantified the rapid decline of UK hedgerows between 1984 and 1990, leading to the Hedgerows Regulations Act of 1997 (The Hedgerows Regulations 1997) to halt the destruction of these important landscape features. Over the years, the GB Countryside Survey has collected a wealth of data that can be flexibly interrogated to address changing policy interest and priorities, for example, reporting on the past trends of condition and extent of newly defined broad habitat categories when they were introduced in 1994 as part of the new UK Biodiversity Action Plan (Firbank et al. 2003), assessing ecosystem services (Smart et al. 2010) and monitoring Natural Capital (Norton et al. 2012b; Norton et al. 2018).

While the GB Countryside Survey data have been maintained as a recognised dataset of national importance with successive surveys comprising ongoing monitoring, nine other datasets were at risk of being lost. These datasets – with a regional, woodland, or other targeted habitat focus – provide valuable records of historic biodiversity, now rescued and safeguarded in the EIDC and additional data publications (Table 1 and the Online Resource). These datasets share the characteristics that have made the GB Countryside Surveys an enduring success (summarised above; Metzger et al. 2013) and were originally designed to detect environmental change.

Following the rescue and publication of the 1971/2001 Woodland Survey data (Wood et al. 2015) and Scottish Pinewood Survey (Wood and Bunce 2016a), the Woodland Trust and other funding bodies supported a new re-survey of the statistically selected sample of 103 broadleaved woods across Britain and 27 native pinewoods in Scotland. Between 2018 and 2022, information was captured for a wide range of ecological parameters, providing an assessment of woodland change across Britain over a 50-year timespan. Some of the recent insights include evidence that ash dieback causes an increase in ground flora species richness and forb cover, herbivory damage by deer is reducing regeneration of trees and shrubs and the impacts of climate change are possibly leading to an increase in holly (*Ilex aquifolium*) (Smart et al. 2024). An assessment of the condition of the Native Pinewood habitat following decades of conservation effort is currently underway (TBC 2024). Having the data, including the plot locations, tree diameter at breast height data and location notes readily at hand meant that the practical re-survey was possible, and could be a key part of the analysis. Having the historic data in a usable format meant that analysis of change could be achieved in a timely manner, providing key information to stakeholders.

The Shetland, Cumbria and ‘Key Habitat’ surveys have not yet been re-surveyed, but the data rescue has made this possible, as demonstrated for the woodland surveys. The ‘Key Habitat’ surveys, focusing on calcareous, coast and heath landscapes, could potentially be integrated into the GB Countryside Surveys, providing additional insight into changes in rarer landscape and habitat types. A repeat of the Shetland survey would provide insights in landscape and vegetation change on the islands following since the rapid economic development following the arrival of the North Sea oil industry and include insight into peatland degradation and biodiversity decline from agricultural intensification (Wood and Bunce 2016b). These datasets provide valuable biodiversity and natural capital baselines that are available for future research (Smart et al. 2024; TBC 2024).

Bob Bunce’s legacy extends beyond the surveys he initiated, and include his efforts to develop a European habitat classification (Bunce et al. 2008), his support and inspiration for ecological monitoring schemes in Sweden, Spain and Austria, his leadership in the International Association for Landscape Ecology in the UK (Young et al. 2020) and internationally, and his mentorship of students and colleagues while working in the UK, The Netherlands, Spain and Estonia (Howard, 2022). But the Bunce surveys provide his most tangible legacy and are now protected and available for future use.

## Conclusion

Rescued data helps to strengthen ecological understanding of biodiversity change. A decade of data rescue and archiving to safeguard data from strategic ecological surveys in the UK has resulted in a structured five-step approach that can benefit other data rescue initiatives as well as scientists planning new ecological monitoring projects. Valuable lessons learnt in the process include: (1) the growing appreciation and relevance of historic ecological data; (2) the importance of adequate resourcing and recognition of data rescue activity; (3) the value of engaging with the originators; (4) the need to identify and understand potential users and uses of the data. The Bunce legacy of strategic ecological surveys in the UK is now protected and the data available for repeat survey and further analysis.

## Supplementary information

Supplementary

## Data Availability

All datasets mentioned in the text have been deposited in the Natural Environment Research Council Environmental Information Data Centre https://eidc.ac.uk/. All datasets have been issued with Digital Object Identifiers, which are cited as appropriate within the text.
