# Supplementary material for: Protecting the Bunce Legacy: Lessons Learned From Safeguarding Long-term Ecological Survey Datasets in Great Britain
Source: Environ Manage. 2025 Apr 25;75(7):1872–85. doi: 10.1007/s00267-025-02175-5 (PMC12228649; doi:10.1007/s00267-025-02175-5)
Supplement: Supplementary file 1 — Supplementary [file 267_2025_2175_MOESM1_ESM.docx]

***Environmental management***

**Protecting the Bunce legacy: lessons learned from safeguarding long-term ecological survey datasets in Great Britain**

Wood, C.M.^1*^, Metzger, M.J.^2^, Bunce, R.G.H.^1,3^

^1^UK Centre for Ecology & Hydrology, Library Avenue, Lancaster, UK. LA1 4AP

^2^ School of GeoSciences, University of Edinburgh, Edinburgh, UK. EH8 9XP

^3^ Estonian University of Life Sciences, Kreuzwaldi 5, 51014 Tartu, Estonia

*Corresponding author [clamw@ceh.ac.uk](mailto:clamw@ceh.ac.uk)

<https://orcid.org/0000-0002-0394-2998>

**Supplementary table:** the Bunce legacy, including published datasets and data papers

| *Date and project* | *Characteristics* | *DOI for data* | *Data paper* |
| --- | --- | --- | --- |
| **1969 Lake District woodlands** | -48 woodlands in the English Lake District  -6 random dispersed vegetation plots per wood  -Details about plant species and trees and shrubs present | <https://doi.org/10.5285/272a131f-7d57-47b4-a643-c3828722d663> | N/A |
| **1971 Woodland Survey** | -2453 woodlands in UK defined Survey cartographically  Limited species lists classified statistically into 103 groups of woodlands  -Analysis of environmental variables from the woodlands  103 sites selected at random from the environmental analysis  -16 random dispersed 200 m square vegetation plots  -Classification using ISA (TWINSPAN) into 13 site classes and 32 plot classes  -Interpretation of the site and plot vegetation classifications | <https://doi.org/10.5285/4d93f9ac-68e3-49cf-8a41-4d02a7ead81a>  https://doi.org/10.5285/fb1e474d-456b-42a9-9a10-a02c35af10d2  https://doi.org/10.5285/d6409d40-58fe-4fa7-b7c8-71a105b965b4  https://doi.org/10.5285/2d023ce9-6dbe-4b4f-a0cd-34768e1455ae | Wood, C.M.; Smart, S.M.; Bunce, R.G.H.. 2015 Woodland Survey of Great Britain 1971-2001. Earth System Science Data, 7 (2). 203-214. https://doi.org/10.5194/essd-7-203-2015 |
| **1971 Scottish Pinewoods Survey** | -27 pinewoods in Scotland  -16 random dispersed 200 m square vegetation plots per wood  -Details about plant species and trees and shrubs present --Interpretation of the plot and site classifications | <https://doi.org/10.5285/56a48373-771c-4d4a-8b5a-45ef496c6e55> | Wood, Claire M.; Bunce, Robert G.H.. 2016 Ecological survey of the native pinewoods of Scotland 1971. Earth System Science Data, 8 (1). 177-189. https://doi.org/10.5194/essd-8-177-2016 |
| **Ecological survey of the Spey Valley birch woods, 1973** | -18 woodlands in Scotland  -16 random dispersed 200 m square vegetation plots per wood  -Details about plant species and trees and shrubs present  -Interpretation of the plot and site classifications | https://doi.org/10.5285/c84961e3-b9dc-4c92-b316-36295b8a3330 | N/A |
| **Regeneration survey from native pinewoods in Scotland, 1973** | -7 pinewoods in Scotland  -16 random dispersed 200 m square vegetation plots per wood  -Details about plant species and trees and shrubs present, and regeneration information  -Interpretation of the plot and site classifications | https://doi.org/10.5285/b2a70d49-893a-406c-802f-1a979eb5013b | N/A |
| **1974 Shetland Ecological Survey** | -Environmental data from 2046 1-km^2^ Ecological Survey squares on a 1 X 1 km grid in Shetland  -Classification by ISA (TWINSPAN) into 16 land classes  -Five random 1-km^2^ squares drawn from each class  -Up to 16 dispersed random 200 m^2^ vegetation plots (per 1km^2^) classified by ISA (TWINSPAN) into 16 vegetation classes  -Interpretation of the characteristics of the land classes and vegetation classes | <https://doi.org/10.5285/f1b3179e-b446-473d-a5fb-4166668da146>    https://doi.org/10.5285/06fc0b8c-cc4a-4ea8-b4be-f8bd7ee25342 | Wood, Claire M.; Bunce, Robert G.H.. 2016 Survey of the terrestrial habitats and vegetation of Shetland, 1974 – a framework for long-term ecological monitoring. Earth System Science Data, 8 (1). 89-103. https://doi.org/10.5194/essd-8-89-2016 |
| **1975 Cumbria Ecological Survey** | -Environmental data from 850 1-km^2^ Ecological Survey squares on a 3 X 3 km grid in Cumbria  -Classification by ISA (TWINSPAN) into 16 land classes  -Remaining 6650 1-km^2^ squares in Cumbria classified by key attributes  -Three random 1-km^2^ squares drawn from each class  -Up to 16 dispersed random 200 m^2^ vegetation plots (per 1km^2^) classified by ISA (TWINSPAN) into 16 vegetation classes  -Interpretation of the characteristics of the land classes and vegetation classes | <https://doi.org/10.5285/e96b909d-d52e-4b36-bc51-905ece420794>    https://doi.org/10.5285/0ac6249c-a6f2-4147-8ae9-50d576e85fc5 | N/A |
| **Cumbria Marginal Uplands 1978** | -52 random 1-km^2^ squares drawn from marginal upland land classes  -Five dispersed random 200 m^2^ vegetation plots (per 1km^2^)  -Interpretation of the characteristics of the land classes and vegetation classes | https://doi.org/10.5285/a4f2d52b-1515-434e-bfc3-83b3a53be1c5 | N/A |
| **1978+**  **Great Britain Countryside Survey** | -Environmental data from 1228 1 km^2^ Great Britain squares on a 15 X 15 km grid in UK Countryside  -Classification by TWINSPAN into 45 Survey land classes  -Allocation by a statistical routine of the remaining c. 233 000 1-km2 squares  -In 1978, 256 1-km^2^ squares drawn at random, increasing progressively to 591 1 km^2^ squares in 2007  -Up to 42 vegetation plots (per 1km^2^), mapping of broad habitats and records of other ecological data  -Classification by TWINSPAN into 100 vegetation classes  -Integrated interpretation of resources and change of broad habitats, vegetation classes and vascular plants | Many – umbrella catalogue link at:  https://catalogue.ceh.ac.uk/documents/2069de82-619d-4751-9904-aec8500d07e6 | Ecological landscape elements: long-term monitoring in Great Britain, the Countryside Survey 1978-2007 and beyond  Wood, Claire M.; Bunce, Robert G.H.; Norton, Lisa R.; Maskell, Lindsay C.; Smart, Simon M.; Scott, W. Andrew; Henrys, Peter A.; Howard, David C.; Wright, Simon M.; Brown, Michael J.; Scott, Rod J.; Stuart, Rick C.; Watkins, John W.. 2018 Ecological landscape elements: long-term monitoring in Great Britain, the Countryside Survey 1978-2007 and beyond. Earth System Science Data, 10 (2). 745-763. https://doi.org/10.5194/essd-10-745-2018  Wood, Claire M.; Smart, Simon M.; Bunce, Robert G.H.; Norton, Lisa R.; Maskell, Lindsay C.; Howard, David C.; Scott, W. Andrew; Henrys, Peter A.. 2017 Long-term vegetation monitoring in Great Britain - the Countryside Survey 1978-2007 and beyond. Earth System Science Data, 9 (2). 445-459. https://doi.org/10.5194/essd-9-445-2017 |
| **1992-93 Survey of ‘Key Habitats’ in England** | -Environmental data from 4 spatial masks in England (Calcareous, Coastal, upland/Lowland Heath)  -213 1-km^2^ squares drawn at random  -Up to 25 vegetation plots per 1-km^2^ square  -Integrated interpretation of resources and change of habitats, vegetation classes and vascular plants | https://doi.org/10.5285/7aefe6aa-0760-4b6d-9473-fad8b960abd4  <https://doi.org/10.5285/dc583be3-3649-4df6-b67e-b0f40b4ec895> | Wood, Claire M.; Bunce, Robert G.H.; Norton, Lisa R.; Smart, Simon M.; Barr, Colin J.. 2018 Land cover and vegetation data from an ecological survey of `key habitat' landscapes in England, 1992-1993. Earth System Science Data, 10 (2). 899-918. https://doi.org/10.5194/essd-10-899-2018 |
